# Impact of immigration status on health behaviors and perceptions in cancer survivors

**DOI:** 10.1002/cam4.2079

**Published:** 2019-03-21

**Authors:** Sophia Y. Liu, Lin Lu, Dan Pringle, Mary Mahler, Chongya Niu, Rebecca Charow, Kyoko Tiessen, Christine Lam, Oleksandr Halytskyy, Hiten Naik, Henrique Hon, Margaret Irwin, Vivien Pat, Christina Gonos, Catherine W. T. Chan, Jodie Villeneuve, Ravi M. Shani, Maha Chaudhry, M. Catherine Brown, Peter Selby, Doris Howell, Wei Xu, Shabbir M. H. Alibhai, Jennifer M. Jones, Geoffrey Liu, Lawson Eng

**Affiliations:** ^1^ Division of Medical Oncology and Hematology, Department of Medicine Princess Margaret Hospital/University Health Network and University of Toronto Toronto ON Canada; ^2^ Department of Biostatistics Princess Margaret Cancer Centre/University Health Network and University of Toronto Toronto ON Canada; ^3^ Ontario Cancer Institute Toronto ON Canada; ^4^ Dalla Lana School of Public Health University of Toronto Toronto ON Canada; ^5^ Centre for Addiction and Mental Health University of Toronto Toronto ON Canada; ^6^ Departments of Family and Community Medicine and Psychiatry University of Toronto Toronto ON Canada; ^7^ Department of Medicine University Health Network and University of Toronto Toronto ON Canada; ^8^ Cancer Rehabilitation and Survivorship Program, Department of Supportive Care Princess Margaret Cancer Centre Toronto ON Canada

**Keywords:** alcohol, cancer survivorship, health behaviors, immigration, patient perceptions, physical activity, smoking

## Abstract

**Background:**

Health behaviors including smoking cessation, physical activity (PA), and alcohol moderation are key aspects of cancer survivorship. Immigrants may have unique survivorship needs. We evaluated whether immigrant cancer survivors had health behaviors and perceptions that were distinct from native‐born cancer survivors.

**Methods:**

Adult cancer patients from Princess Margaret Cancer Centre were surveyed on their smoking, PA, and alcohol habits and perceptions of the effects of these behaviors on quality of life (QoL), 5‐year survival, and fatigue. Multivariable models evaluated the association of immigration status and region‐of‐origin on behaviors and perceptions.

**Results:**

Of the 784 patients, 39% self‐identified as immigrants. Median time of survey was 24 months after histological diagnosis. At baseline, immigrants had trends toward not meeting Canadian PA guidelines or being ever‐drinkers; patients from non‐Western countries were less likely to smoke (aOR_current _= 0.46, aOR_ex‐smoker _= 0.47, *P* = 0.02), drink alcohol (aOR_current _= 0.22, aOR_ex‐drinker _= 0.52, *P* < 0.001), or meet PA guidelines (aOR = 0.44, *P* = 0.006). Among immigrants, remote immigrants (migrated ≥40 years ago) were more likely to be consuming alcohol at diagnosis (aOR = 5.70, *P* < 0.001) compared to recent immigrants. Compared to nonimmigrants, immigrants were less likely to perceive smoking as harmful on QoL (aOR = 0.58, *P* = 0.008) and survival (aOR = 0.56, *P* = 0.002), and less likely to perceive that PA improved fatigue (aOR = 0.62, *P* = 0.04) and survival (aOR = 0.64, *P* = 0.08).

**Conclusions:**

Immigrants had different patterns of health behaviors than nonimmigrants. Immigrants were less likely to perceive continued smoking as harmful and were less likely to be aware of PA benefits. Culturally tailored counselling may be required for immigrants who smoke or are physically sedentary at diagnosis.

## 1 INTRODUCTION

In 2009, the number of cancer survivors in Canada reached over 810 000, representing about 2.4% of the Canadian population.^1^ Given advances in early cancer detection and improvement in cancer treatment options,^2^ this survivorship population is expected to grow. As a result, secondary prevention measures (to reduce recurrence and second primary risk), managing long‐term toxicities, and optimizing quality of life are becoming important priorities in cancer care.

Health behaviors, such as smoking cessation, routine physical activity (PA), and alcohol moderation, can help reduce risk of both recurrence and second primaries, while also improving quality of life in some cancers.^3^, ^4^, ^5^, ^6^ Improving health behaviors can impact not only cancer outcomes, but also help manage other common comorbidities such as cardiovascular disease.^7^

In recent years, the number of global migrants has continued to grow, reaching 258 million in 2017. Higher income countries have had the most growth in global migrants, hosting 64% of all international migrants in 2017.^8^ In Canada, approximately one in five individuals are foreign‐born; this proportion is projected to increase to 30.0% by 2036.^9^ Disparities in cancer care between immigrant and native‐born patients have been reported across the entire cancer control continuum: from cancer screening, receiving treatment recommendations, mortality rates, to survivorship issues such as psychosocial well‐being and quality of life.^10^, ^11^, ^12^, ^13^, ^14^ The care gap between immigrant and native‐born cancer survivors is becoming increasingly important, given the changing demographics of many developed countries.

Recently, lower cancer‐specific mortality for immigrants has been reported, when compared to nonimmigrants.^15^ The healthy immigrant advantage diminished with successive years spent in Canada; this is thought to be influenced in part by health behavior changes.^15^ Given the healthy immigrant effect and discrepancies in cancer care,^10^, ^11^, ^12^, ^13^, ^14^ there is a greater need to better manage the long‐term survivorship needs of this growing population. Survivorship studies on immigrant care have focused previously on survivorship care plans, disparities in quality of life, and perceived cancer care.^12^, ^16^, ^17^ However, differences in both health behaviors and perceptions of these behaviors between immigrant and native‐born cancer survivors have not been explored. Previously, our group demonstrated that patient perceptions can be associated with health behavior changes after a cancer diagnosis.^18^, ^19^, ^20^

Our study objective was to identify potential areas requiring special considerations for survivorship programming in immigrant cancer survivors. Our specific aims are as follows: to compare immigrant and native cancer survivors for smoking, PA, and alcohol consumption patterns before and after diagnosis (Aim 1) and perceptions of these behaviors on survivorship outcomes (Aim 2). Among immigrants, we compared both behaviors and their perceptions between recent (migrated to Canada <40 years ago) and remote (≥40 years ago) immigrants.

## 2 PATIENTS AND METHODS

### 2.1 Patient recruitment and data collection

Adult cancer survivors (defined as any individual aged 18 years or over who is carrying a diagnosis of cancer at any point in his or her lifetime) from all disease sites were surveyed from ambulatory oncology clinics between 2012 and 2014 with a one‐time questionnaire at a tertiary cancer center, Princess Margaret Cancer Centre (Toronto, ON, Canada), whose institutional research ethics board also approved the study. Patients with a histologic confirmation of malignancy (solid or hematologic) of all stages and treatment intents were included. Patients with cognitive and language proficiency deficiencies preventing patient understanding of the study or affecting the ability to provide consent were excluded from the study. Patients diagnosed with cancer more than 10 years before the recruitment date were excluded because we wanted to focus on patients who still required more frequent oncology follow‐up.

All consenting patients completed a one‐time, self‐administered and self‐reported questionnaire at an ambulatory oncology clinic visit, assessing socio‐demographics, including country of birth and year moved to Canada, functional status (measured on the Eastern Cooperative Oncology Group (ECOG) scale and separately on a 5‐point Likert scale ranging from Poor to Excellent), smoking exposure, alcohol consumption, and PA histories and perceptions of these behaviors. Clinico‐pathologic variables including date of histologic diagnosis, tumor site, cancer stage, and cancer treatment data were obtained through patient medical record review.

### 2.2 Assessment of outcomes

Smoking, alcohol, and PA histories were classified similar to our prior studies.^18^, ^19^, ^20^, ^21^ Patients were asked to self report their smoking history through a series of questions asking about lifetime cigarette use, duration and intensity of use (ie, number of years smoked and packs smoked per day), quit dates, and current smoking status. Cumulative smoking history was calculated using pack‐years (number of years smoked × number of cigarette packs smoked per day). Never smokers were defined as having smoked fewer than 100 cigarettes in their lifetime. Patients with a positive smoking history were further subclassified as either current smokers at diagnosis (defined as individuals who smoked within 1 year before their cancer diagnosis) or were otherwise categorized as ex‐smokers. The time frame of the current smoker definition is consistent with our prior studies, which attempts to minimize the confounding effects from cancer symptoms, workup, or staging on behaviors.

Physical activity (PA) levels were assessed using the Godin‐Shephard Leisure‐Time Physical Activity questionnaire,^22^ and classified based on meeting or not meeting Canadian PA guideline recommendations of at least 150 minutes of moderate‐to‐vigorous physical activity (MVPA) per week.^23^ Activity levels were compared between 1 year prior to diagnosis (baseline) and at follow‐up (time of survey). Change in MVPA levels was classified as either maintained or improved to recommended levels at follow‐up, or as reduced to or persisted below recommended levels.^18^

Patients were asked to self‐report their alcohol consumption both at diagnosis and currently based on number of standard drinks per week with a standard alcohol drink guide. Patients were ever‐drinkers if they reported consuming at least one standard drink of alcohol based on 13.6 g of ethanol (5 oz of wine, 12 oz of beer, and 1.5 oz of liquor) per month for one year.^24^ Current drinkers were still consuming at least one standard drink of alcohol per week at the time of cancer diagnosis; ex‐drinkers were not. Current drinkers were further classified based on their level of alcohol consumption at one year after cancer diagnosis (or at follow‐up, if less than 1 year of follow‐up had occurred at the time of survey) as either quitters or those who had cut down on use; others were classified as continued drinkers or those who increased alcohol consumption. Ex‐drinkers were divided between restarters and abstainers at one year after diagnosis.

We assessed patient perceptions on three survivorship outcomes through a brief screening tool used by our group previously. For each health behavior, patients were asked to rate the perceived impact of each behavior on three survivorship outcomes: quality of life, 5‐year overall survival, and fatigue after a cancer diagnosis using a 7‐point Likert scale (1 = “make much worse”, 4 = “no effect”, to 7 = “make much better”).^18^, ^19^, ^20^, ^21^

### 2.3 Definition of immigrants

An *immigrant* was defined as those not born in Canada, and *nonimmigrants* were born in Canada, similar to prior Canadian studies investigating the impact of immigration status in cancer outcomes or health behavior.^15^, ^25^ Immigrants were further subcategorized into *recent immigrants *(those who migrated to Canada <40 years prior to date of recruitment) and *remote immigrants *(those who migrated to Canada ≥40 years prior to date of recruitment). The remote and recent definitions were chosen to allow for adequate sample size in both groups. All participants were also classified geographically based on country of birth as either of Western origin (Canada, United States, and all of Europe) or non‐Western. This dichotomization was used to investigate the effect of originating from a culturally Western dominant country and as part of a sensitivity analysis.

### 2.4 Statistical analyses

All statistical analyses were performed using R statistical software (http://CRAN.R-project.org). Frequencies of socio‐demographic and clinico‐pathologic variables were compared between immigrant (not born in Canada) and native‐born survivors using *t* and *χ* ^2^ tests as appropriate. Univariable analysis was applied to assess the effect of immigration status and other covariates (Table 1), including cumulative pack‐years smoked, minutes of weekly PA at baseline, average drinks per week at diagnosis, income, and education on outcomes using logistic regression. For outcomes where immigration status was significantly associated in univariable analysis (*P* < 0.05), multivariable models were then applied to evaluate the independent effect of immigration status on these outcomes. Co‐variates significant at *P* < 0.10 were included in a multivariable model, where a backward selection algorithm was applied, eliminating nonsignificant covariates (*P* > 0.05) to identify independent variables for each base model. For each base model, immigration status was then added and tested for its significance using the Wald test. A value of *P* < 0.05 was considered significant.

**Table 1:** Socio‐demographic and clinico‐pathologic characteristics of our included study participants stratified by immigrant status. *P* values represent comparisons between immigrant patients and nonimmigrant patients

Variable	Subgroup	Total patients, n = 784 (%)	Immigrant patients, n = 309 (%)	Nonimmigrant patients, n = 475 (%)	*P* value
Socio‐demographic variables					
Gender	Male	47%	48%	46%	0.61
Age at diagnosis	Median (range)	56 (18‐97)	58 (18‐91)	56 (18‐97)	0.07
Follow‐up time	Median (range)	24 (0‐120)	25 (0‐120)	23 (0‐120)	0.18
Language at home	English	91%	79%	100%	<0.001
Employment status	Employed/Equivalent	40%	36%	42%	0.07
Employment type	White collar job	71%	72%	71%	0.87
Marital status	Married/common‐law	72%	73%	71%	0.68
Education	Received any post‐secondary studies	72%	75%	71%	0.22
Household income	≥ $100,000/year	39%	34%	42%	0.02
Self‐rated health	Very good to excellent	35%	29%	39%	0.004
ECOG	0	47%	44%	49%	0.16
Immigration date	Remote (≥40 years ago)	—	46%	—	—
Immigration region	Western	—	57%	—	—
Clinico‐pathological variables					
Disease stage	Localized	73%	71%	74%	0.67
	Metastatic	10%	11%	9%	
	Hematologic	17%	18%	16%	
Treatment intent at diagnosis	Curative	80%	77%	81%	0.32
Treatment intent at follow‐up (survey time)	Curative	69%	65%	71%	0.16
Systemic therapy	Received	62%	65%	60%	0.13
Radiation therapy	Received	47%	47%	47%	0.88
Surgery	Received	60%	61%	59%	0.71

## 3 RESULTS

### 3.1 Baseline socio‐demographic and clinico‐pathologic variables

**Figure 1:**
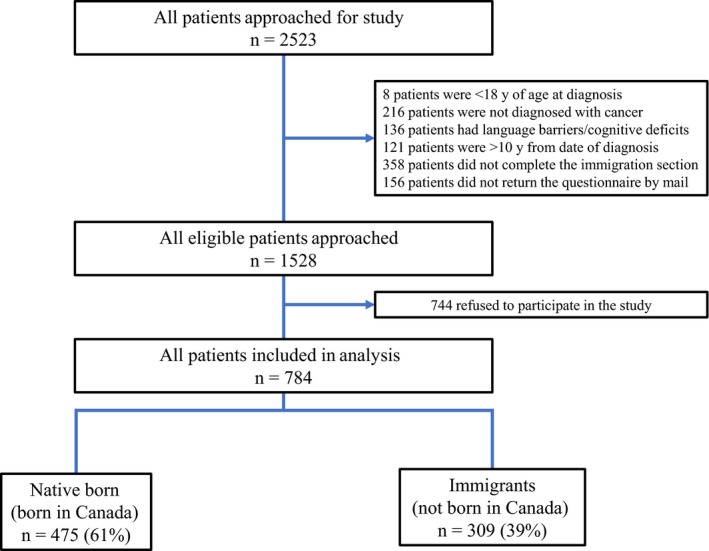
Recruitment statistics and distribution of patients approached and enrolled in study based on immigration status and among immigrants, distribution of recent and remote immigrants. In this study, an immigrant is defined as someone not born in Canada and native‐born individuals were born in Canada. Recent immigrant is defined as someone who has resided in Canada <40 y, and remote if resided in Canada ≥40 y

**Figure 2:**
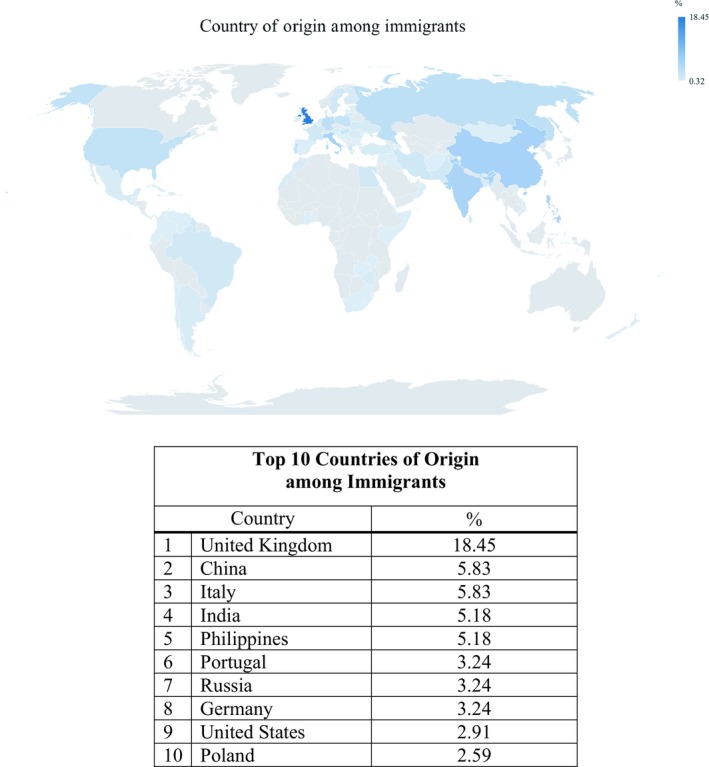
Geographical distribution and country of origin among immigrant cancer patients by percentage of total

Of the 2523 patients approached, 1528 were eligible and 784 participated in the study. The effective response rate was 51%. Median follow‐up time was 24 months. Recruitment statistics and distribution by immigration status are presented in Figure 1. There was no difference in age (*P* = 0.66) or gender (*P* = 0.17) between responders and nonresponders, although responders were more likely to report very good to excellent health compared to nonresponders (35% vs 27%, *P* = 0.001). The socio‐demographic and clinico‐pathological characteristics of our study population are shown in Table 1. Among all patients recruited, 39% were immigrants, with 54% of immigrants being recent immigrants and 43% of all patients were of non‐Western origin. Among all patients, the majority were English speaking, married or in a common‐law marriage, post‐secondary graduates, not currently employed, and relatively asymptomatic from their disease. Most patients had localized disease and received curative‐intent therapy at diagnosis. Immigrants were more likely to speak a non‐English language at home, have a household income below $100 000 per year, and have poorer self‐rated health. Median age at diagnosis and education were not significantly different compared to nonimmigrants. Recruited patients represented a broad disease site distribution including breast (16%), gastrointestinal (12%), genitourinary (13%), gynecologic (12%), head and neck (11%), hematologic (17%), lung (8%), and other (11%) cancers. The distribution of cancer patients’ country of origin is shown in Figure 2.

### 3.2 Smoking

The distribution of patients based on smoking status and perceptions of smoking on survivorship outcomes stratified by immigration status is shown in Table 2. During the peri‐diagnosis period, 16% were smoking and approximately half were lifetime never smokers. Of current smokers, 49% had quit after their cancer diagnosis.

**Table 2:** Comparison of behavior changes (smoking, PA and alcohol moderation) and perceptions of these behaviors between immigrant and nonimmigrant cancer survivors

Variable	Subgroup	Total patients, n = 784 (%)	Immigrant patients (not born in Canada), n = 309 (%)	Nonimmigrant patients (born in Canada), n = 475 (%)	*P* value
Smoking					
Baseline smoking status	Current smoker	16%	14%	18%	0.08
	Ex‐smoker	31%	29%	33%	
	Never smoker	52%	57%	49%	
Change in smoking status after diagnosis	Continued	51%	39%	57%	0.084
	Quit	49%	61%	43%	
Perception of smoking on quality of life	Worsens outcome	79%	73%	82%	0.008
Perception of smoking on overall survival	Worsens outcome	75%	68%	79%	0.002
Perception of smoking on fatigue	Worsens outcome	72%	69%	74%	0.12
Physical activity					
Baseline PA levels	Meeting MVPA Guidelines	31%	26%	34%	0.02
Change in PA levels among those inactive at baseline	Improved to meeting at MVPA guidelines	12%	13%	11%	0.44
Change in PA levels among those active at baseline	Continued to meet MVPA guidelines	49%	45%	50%	0.53
Perception of PA on quality of Life	Improves outcome	91%	88%	92%	0.06
Perception of PA on overall survival	Improves outcome	89%	84%	91%	0.006
Perception of PA on fatigue	Improves outcome	77%	70%	82%	0.001
Alcohol					
Alcohol use status at diagnosis	Current drinker	59%	47%	66%	<0.001
	Ex‐drinker	17%	17%	16%	
	Never drinker	25%	36%	18%	
Change in alcohol use for Current Drinkers at diagnosis	Quit/Cut down	52%	50%	53%	0.66
	Increased/Continued	48%	50%	47%	
Change in alcohol use for Ex‐Drinkers at diagnosis	Restarted	21%	21%	20%	1
	Remained abstinent	79%	79%	80%	
Perception of alcohol on quality of life	Worsens outcome	38%	42%	35%	0.07
Perception of alcohol on overall survival	Worsens outcome	38%	41%	36%	0.25
Perception of alcohol on fatigue	Worsens outcome	45%	47%	44%	0.42

*P* values represent comparisons between immigrant and nonimmigrant cancer survivors. *Change in smoking status was assessed only for current smokers; no ex‐smokers restarted and no never smokers started smoking after diagnosis.

In multivariable analysis, baseline smoking status did not significantly differ based on immigration status (Table 3). Patients from non‐Western countries were less likely to be current (aOR = 0.46; 95% CI [0.20‐1.04]); *P* = 0.06) or ex‐smokers at baseline (aOR = 0.47; 95% CI [0.25‐0.87]); *P* = 0.02) compared to never smokers (Table 4), which likely contributed to a similar nonsignificant univariable trend (*P* = 0.08) for immigrants (Table 3). Quit rates did not significantly differ based on immigration status or country of origin (Tables 3 and 4). Baseline smoking status or quit rates were also not different based on time of immigration (Table 5).

**Table 3:** Univariable and multivariable analysis of the impact of immigration status (immigrant vs nonimmigrant) on significant outcomes in univariable analysis for smoking, PA, and alcohol

Specific outcome	Outcome comparison	Comparison of immigrant vs nonimmigrant cancer survivors on specific outcomes				Adjustment variables in final model
		Univariable analysis		Multivariable analysis		
		OR (95% CI)	*P*	aOR (95% CI)	*P*	
Smoking						
Baseline smoking status	Current vs Never Smoker	0.66 (0.43‐0.99)	0.08	—	—	—
	Ex‐Smoker vs Never Smoker	0.77 (0.55‐1.06)		—		
Change in smoking status after diagnosis among current smokers at diagnosis	Quit vs Continued	2.05 (0.95‐4.42)	0.07	—	—	—
Perception of smoking on quality of life	Worsens vs No Effect/Improves	0.59 (0.41‐0.86)	0.006	0.58 (0.39‐0.86)	0.008	a
Perception of smoking on overall survival		0.56 (0.39‐0.80)	0.002	0.56 (0.39‐0.80)	0.002	—*
Perception of smoking on fatigue		0.76 (0.53‐1.07)	0.12	—	—	b
Physical activity						
Baseline PA levels	Exercising at MVPA levels vs not	0.67 (0.48‐0.94)	0.02	0.70 (0.47‐1.04)	0.08	b, c, d
Change in PA levels among those inactive at baseline	Improved to Exercising at MVPA levels vs not	1.28 (0.71‐2.32)	0.41	—	—	—
Change in PA levels among those active at baseline	Continued Exercising at MVPA levels vs not	0.80 (0.43‐1.49)	0.48	—	—	—
Perception of PA on Quality of Life	Improves vs No Effect/Worsens	0.6 (0.36‐1.00)	0.05	1.41 (0.66‐3.03)	0.38	c, e, f
Perception of PA on overall survival		0.51 (0.32‐0.81)	0.005	0.64 (0.39‐1.05)	0.08	d, f, g
Perception of PA on Fatigue		0.54 (0.37‐0.77)	<0.001	0.62 (0.40‐0.97)	0.04	c, e, h
Alcohol						
Alcohol use status at diagnosis	Current vs Never Drinker	0.37 (0.25‐0.52)	<0.001	0.58 (0.36‐0.94)	0.08	c, i, j
	Ex‐Drinker vs Never Drinker	0.52 (0.32‐0.83)		0.58 (0.31‐1.09)		
Change in alcohol use among current drinkers at diagnosis	Quit/Cut down vs. Continued/increased	1.11 (0.72‐1.72)	0.62	—	—	—
Perception of alcohol on quality of life	Worsens vs No Effect/Improves	1.34 (0.98‐1.84)	0.07	—	—	—
Perception of alcohol on overall survival		1.22 (0.89‐1.69)	0.22	—	—	—
Perception of alcohol on fatigue		1.15 (0.84‐1.57)	0.39	—	—	—

Each final base model of outcome was adjusted differently for variables in far‐right column: a) Pack‐years smoked b) Education c) Income d) Radiation Treatment e) Self‐Rated Health f) Baseline minutes of MVPA g) Age at diagnosis h) Employment Type i) Gender j) Language.

* For this outcome, there were no significant co‐variates found associated with the outcome. These variables were selected using backward selection modelling from all socio‐demographic and clinico‐pathological variable (Table 1) and relevant behavior variables (Table 2) that were significant with that outcome.

**Table 4:** Univariable and multivariable analysis of impact of world region of origin (non‐western vs western) on significant outcomes in univariable analysis for smoking, PA, and alcohol

Specific outcome	Outcome subgroup	Comparison of cancer survivors from non‐western vs western country of origin on specific outcomes				Adjustment variables in final model
		Univariable analysis		Multivariable analysis		
		OR (95% CI)	*P*	aOR (95% CI)	*P*	
Smoking						
Baseline smoking status	Current vs never smoker	0.56 (0.31‐0.99)	0.003	0.46 (0.20‐1.04)	0.02	a, b, c, d, e
	Ex‐smoker vs never smoker	0.48 (0.30‐0.76)		0.47 (0.25‐0.87)		
Change in smoking status after diagnosis among current smokers at diagnosis	Quit vs continued	1.94 (0.66‐5.74)	0.23	—	—	—
Perception of smoking on quality of life	Worsens vs no effect/improves	0.51 (0.32‐0.81)	0.004	0.50 (0.30‐0.81)	0.005	f
Perception of smoking on overall survival		0.64 (0.40‐1.01)	0.05	0.64 (0.40‐1.01)	0.05	—*
Perception of Smoking on Fatigue		0.71 (0.45‐1.12)	0.14	—	—	—
Physical Activity						
Baseline PA levels	Exercising at MVPA levels vs not	0.48 (0.30‐0.78)	0.003	0.44 (0.25‐0.79)	0.006	b, d, g
Change in PA levels among those inactive at baseline	Improved to Exercising at MVPA levels vs not	1.21 (0.59‐2.49)	0.60	—	—	—
Change in PA levels among those active at baseline	Continued Exercising at MVPA levels vs not	0.46 (0.17‐1.27)	0.14	—	—	—
Perception of PA on quality of life	Improves vs no effect/worsens	0.72 (0.38‐1.35)	0.30	—	—	—
Perception of PA on overall survival		0.87 (0.47‐1.61)	0.66	—	—	—
Perception of PA on fatigue		0.68 (0.43‐1.08)	0.10	—	—	—
Alcohol						
Alcohol use status at diagnosis	Current vs never drinker	0.16 (0.10‐0.26)	<0.001	0.22 (0.12‐0.40)	<0.001	b, h, i
	Ex‐drinker vs never drinker	0.51 (0.30‐0.88)		0.52 (0.26‐1.05)		
Change in alcohol use among current drinkers at diagnosis	Quit/cut down vs. Continued/increased	0.76 (0.36‐1.61)	0.48	—	—	—
Perception of Alcohol on Quality of Life	Worsens vs no effect/improves	2.08 (1.39‐3.13)	<0.001	1.47 (0.95‐2.27)	0.08	a, j, k
Perception of alcohol on overall survival		1.72 (1.12‐2.63)	0.01	1.22 (0.78‐1.92)	0.39	a, j, k
Perception of alcohol on fatigue		1.67 (1.11‐2.56)	0.02	1.27 (0.81‐1.96)	0.29	a, h, j, k

Each final base model of outcome was adjusted differently for variables in far‐right column: a) Age at diagnosis, b) Income c) Employment Type d) Education e) Self‐Rated Health f) Pack Years g) Radiation Treatment h) Gender i) Language j) Number of drinks at diagnosis k) ECOG.

* For this outcome, there were no significant co‐variates found associated with the outcome. These variables were selected using backward selection modelling from all socio‐demographic and clinico‐pathological variable (Table 1) and relevant behavior variables (Table 2) that were significant with that outcome.

**Table 5:** Univariable and multivariable analysis of impact of remote vs recent immigrant dichotomization on significant outcomes in univariable analysis for smoking, PA, and alcohol use and perceptions toward these behaviors

Specific outcome	Outcome subgroup	Comparison of remote vs recent immigrant cancer survivors on specific outcomes				Adjustment variables in final model
		Univariable analysis		Multivariable analysis		
		OR (95% CI)	*P*	aOR (95% CI)	*P*	
Smoking						
Baseline smoking status	Current vs never smoker	1.50 (0.73‐3.08)	0.39	—	—	—
	Ex‐smoker vs never smoker	1.34 (0.79‐2.28)		—		
Change in smoking status after diagnosis among current smokers at diagnosis	Quit vs continued	0.30 (0.07‐1.27)	0.10	—	—	—
Perception of smoking on quality of life	Worsens vs no effect/improves	1.56 (0.87‐2.79)	0.13	—	—	—
Perception of smoking on overall survival		1.25 (0.71‐2.19)	0.43	—	—	—
Perception of smoking on fatigue		1.40 (0.79‐2.47)	0.24	—	—	—
Physical activity						
Baseline PA levels	Exercising at MVPA levels vs not	0.68 (0.39‐1.19)	0.18	—	—	—
Change in PA levels among those inactive at baseline	Improved to Exercising at MVPA levels vs not	0.90 (0.36‐2.21)	0.81	—	—	—
Change in PA levels among those active at baseline	Continued Exercising at MVPA levels vs not	0.57 (0.19‐1.72)	0.32	—	—	—
Perception of PA on quality of life	Improves vs no effect/worsens	1.24 (0.59‐2.64)	0.57	—	—	—
Perception of PA on overall survival		0.68 (0.34‐1.36)	0.28	—	—	—
Perception of PA on fatigue		1.04 (0.60‐1.80)	0.88	—	—	—
Alcohol						
Alcohol use status at diagnosis	Current vs never drinker	4.95 (2.70‐9.10)	<0.001	5.70 (2.41‐13.49)	<0.001	a, b, c
	Ex‐drinker vs never drinker	2.20 (1.00‐4.84)		4.21 (1.37‐12.86)		
Change in alcohol use among current drinkers at diagnosis	Quit/cut down vs. continued/increased	1.10 (0.52‐2.32)	0.81	—	—	—
Perception of alcohol on quality of life	Worsens vs no effect/improves	0.42 (0.25‐0.72)	0.002	0.69 (0.37‐1.31)	0.26	d, e, f
Perception of alcohol on overall survival		0.38 (0.21‐0.66)	<0.001	0.62 (0.32‐1.22)	0.17	d, e, f
Perception of alcohol on fatigue		0.51 (0.30‐0.86)	0.01	0.76 (0.40‐1.43)	0.40	a, d, e, f

Each final base model of outcome was adjusted differently for variables in far‐right column: a) Gender b) Income c) Language d) Age at diagnosis e) Number of drinks at diagnosis f) ECOG. These variables were selected using backward selection modelling from all socio‐demographic and clinico‐pathological variable (Table 1) and relevant behavior variables (Table 2) that were significant with that outcome.

Although more than 72% of all patients perceived smoking to be harmful on quality of life, overall survival, and fatigue, immigrants were less likely to perceive smoking as harmful on quality of life (aOR = 0.58; 95% CI [0.39‐0.86]; *P* = 0.008) and survival (aOR = 0.56; 95% CI [0.39‐0.80]; *P* = 0.002) (Table 3). Similarly, cancer survivors of non‐Western origin had similar results, with an aOR = 0.50; 95% CI [0.30‐0.81], *P* = 0.005 for quality of life and aOR = 0.64; 95% CI [0.40‐1.01], *P* = 0.05 for survival (Table 4). There were no significant differences in perceptions based on time of immigration (Table 5).

### 3.3 Physical activity

The distribution of PA levels and perceptions of them on survivorship outcomes are shown in Table 2. Among all patients, only 31% met MVPA guidelines one year before diagnosis, and half (49%) continued to meet MVPA guidelines at follow‐up. Of those who did not meet guidelines at baseline (69%), only 12% improved to meeting PA guidelines at any point during cancer treatment.

In multivariable analysis, there was a nonsignificant trend toward immigrants not meeting PA guidelines (aOR = 0.70; 95% CI [0.47‐1.04]; *P* = 0.08) at baseline (Table 3). Patients from non‐Western countries were half as likely to meet PA guidelines at baseline (aOR = 0.44; 95% CI [0.25‐0.79]; *P* = 0.006) (Table 4), likely contributing to the trend for all immigrants. No associations were found for change in PA levels after diagnosis based on immigration status or country of origin. No differences in baseline or change in PA levels were found based on time of immigration (Table 5).

The majority of patients believed that PA improved fatigue (77%), overall survival (89%), and quality of life (91%). Immigrants were a third less likely to perceive that PA improves fatigue (aOR = 0.62; 95% CI [0.40‐0.97]; *P* = 0.04) and overall survival (aOR = 0.64; 95% CI [0.39‐1.05]; *P* = 0.08) (Table 3), although the latter trend was nonsignificant. No differences were found based on either country of origin or time of immigration for perceptions of PA (Tables 4 and 5).

### 3.4 Alcohol consumption

Approximately two‐thirds (59%) of patients surveyed consumed alcohol regularly at diagnosis; almost one‐fifth (17%) were ex‐drinkers, and a quarter (25%) were never‐drinkers (Table 2). Of current drinkers, approximately half (52%) had quit or cut down their consumption after diagnosis. Among ex‐drinkers, approximately one‐fifth (21%) restarted while no never‐drinkers started drinking.

In multivariable analysis, immigrants were less likely to be current (aOR = 0.58; 95% CI [0.36‐0.94]; *P* < 0.05) or ex‐drinkers (aOR = 0.58; 95% CI [0.31‐1.09]; *P* = 0.08) at baseline (Table 3). A similar trend (aOR_current _= 0.22; 95% CI [0.12‐0.40]; *P* < 0.001; aOR_ex‐drinker _= 0.52; 95% CI [0.26‐1.05]; *P* < 0.07) was seen for patients of non‐Western origins (Table 4). The timing of immigration was important for alcohol use: when compared to recent immigrants, remote immigrants were five‐fold more likely to be current drinkers (aOR = 5.70; 95% CI [2.41‐13.49], *P* < 0.001) or four‐fold more likely to be ex‐drinkers (aOR = 4.21; 95% CI [1.37‐12.86], *P* < 0.001) (Table 5). No differences for change in alcohol use pattern were found based on immigration status, country of origin, or time of immigration (Table 3, 4, 5).

Less than half the patients perceived that alcohol worsened quality of life (38%), fatigue (38%), or survival (45%). There were no differences based on immigration status or time of immigration with respect to their perceptions of continued alcohol consumption on cancer outcomes (Tables 3 and 5). Cancer survivors from non‐Western countries were more likely to perceive alcohol as harmful on quality of life (aOR = 1.47; 95% CI [0.95‐2.27], *P* = 0.08) (Table 4).

## 4 DISCUSSION

In a large cohort of cancer survivors, we identified that immigrant cancer survivors, and specifically, survivors from non‐Western countries, were less likely to perceive smoking as worsening survival and quality of life. Immigrants were also less likely to perceive PA as beneficial for fatigue and survival. Disparities in behaviors were also found. Patients from non‐Western countries were less likely to be ex‐smokers, physically active, or current drinkers at baseline. Comparing all immigrants to nonimmigrants, there were similar non‐significant trends for tobacco and PA. Among immigrants, alcohol use behavior significantly differed based on time of immigration, with remote immigrants more likely to be current or ex‐drinkers at baseline. Taken together, immigrant cancer survivors, especially those of non‐Western origins, may need specialized delivery of cancer survivorship care on health behaviors.

Prior studies in cancer and noncancer patients have evaluated for differences in health behaviors between immigrants and nonimmigrants. In the general population, lower levels of PA and smoking exist among foreign compared to Canadian‐born individuals.^25^, ^26^ Among cancer survivors, race was previously not found to be associated with being a current smoker, though more white breast cancer survivors were former smokers at diagnosis.^27^ Racial differences were also not identified among survivors for PA, but white breast cancer survivors reported heavy alcohol drinking. Consistent with our results, cancer survivors from visible minorities have been previously identified as being less physically active.^28^ However, among these studies, patients were defined by race and not immigration status.

Identifying survivors as immigrants may be more relevant than identification by race because of unique stresses immigrants face including language barriers, separation from family, cultural isolation, and the acculturation process.^29^ Cancer survivorship research on immigrant health has focused on other areas including quality of life, psychosocial well‐being, and perceived cancer care.^12^, ^16^ Research on immigrant survivor health behaviors have focused on their perceptions on cancer screening or vaccinations.^30^, ^31^ However, to date, no prior study has directly examined the impact of immigration status on health behaviors and perceptions toward these behaviors among cancer survivors. As seen in prior studies by our group, perceptions of these health behaviors may influence behavior change after a cancer diagnosis.^18^, ^19^, ^20^

The differences in baseline health behaviors and perceptions of them on outcomes are likely multifactorial. First, health behaviors and perceptions of them can be influenced by patient knowledge of their diagnosis and awareness of the impact of health behaviors. Immigrants have been found to have poorer knowledge of breast cancer and perceive more barriers to mammography when compared to native women.^30^ Similarly, among Haitian immigrants, there is lack of awareness of the association of HPV and cervical cancer and HPV vaccine educational information.^31^ Up to 16% of immigrants have also been found to be unaware of their correct cancer diagnosis.^32^ This lack of knowledge and awareness may impact both baseline health behaviors leading up to diagnosis and subsequent behavior changes afterward. Second, immigrant cancer patients report greater difficulty communicating with physicians.^16^ Language barriers can limit patient counselling, however, clinicians more frequently use untrained ad hoc interpreters when communicating despite benefits of professional interpreters and self‐identifying communication difficulties as a key barrier for immigrant care.^33^, ^34^ In the non‐cancer setting, clinicians are less likely to counsel on health behaviors when presented with language discordance with patients and patients with limited English proficiency are less likely to receive counselling on PA.^35^, ^36^ Third, cultural differences and stereotypes may influence counselling by clinicians. Doctors may harbor less affiliate feelings toward patients of other cultures, draw stereotypic assumptions regarding risk behaviors and advice compliance which may lead to less counselling on health behaviors.^37^, ^38^ Lastly, cultural stigma and lack of appropriate services among immigrant survivors may limit participation in social programs that may offer survivorship support and education.^39^

Immigrants face many barriers when interacting with the cancer care team including communication difficulties, interpreter issues, cultural isolation, and alienation.^40^ This may, in turn, impact the ability for clinicians to provide survivorship counselling on health behaviors. Our results highlight the need to address immigrants’ perceptions of the harms of smoking and physical inactivity and suggest that immigrant tailored interventions may be required for immigrants who smoke or are sedentary. Potential strategies to address these barriers and provide culturally oriented programs include: (a) designing culturally relevant health behavior interventions targeting immigrant survivors’ behaviors, especially since decisions like tobacco use appear to be largely influenced by cultural values^41^; (b) implementing bilingual cultural navigators and health educators to provide tailored survivorship outreach as similar interventions have helped increase cancer screening among minorities and immigrants^42^, ^43^; (c) integrating medically trained in‐person interpreters into routine clinical practice, for example, perhaps by training university students as medical interpreters. Although telephone interpretation is commonly used, clinicians are more likely to understand patients’ cultural beliefs with in‐person interpreters when compared with video interpretation^44^; (d) investing in cultural competency training of healthcare professionals to improve communication with limited English proficiency patients^45^; (e) improving the cultural sensitivity and access to language specific cancer survivorship material can help improve behavior change as the cultural sensitivity of education materials has been identified as a weakness to address.^46^ An important time to intervene may be the peri‐diagnosis period as it represents a time‐sensitive “teachable moment” for clinicians to influence behavior change and survivors may be highly motivated toward health promotion.^47^

Our study has limitations. First, defining immigrants based on place of birth included patients from countries such as the United States and those who immigrated to Canada at a young age. These patients may be more similar to a native‐born individual as they come from countries with similar behavior patterns or had more time adapting in the new culture, as suggested by alcohol use patterns. However, this limitation may only reduce observed differences among immigrants and over half of our patients had immigrated more recently (as defined by individuals who immigrated within the past 40 years to Canada). Immigrants whose English proficiency prevented understanding or consent to the study were also excluded, leaving out a group whose behaviors and perceptions may further contrast with those of native‐born patients. Second, our effective response rate is below the 60% benchmark for low probability of nonresponse bias as outlined by Dillman et al^48^ which may affect the generalizability of the study. Our results were also from a single, large, tertiary Canadian institution, and the immigration patterns, socio‐economic integration process, and universal health care system of Canada may further influence the generalizability to other jurisdictions, as other countries may have different recommendations related to secondary prevention and survivorship care. Similarly, among non‐Western countries, there may be differences based on region of origin which can influence results and further studies evaluating large groups from the same region will be required to have a better understanding of specific regional group differences. Third, smoking and alcohol consumption were assessed and classified as per our previous studies, and our assessment tool for perceptions of each behavior's impact on quality of life, survival, and fatigue was exploratory. Further validation of these items’ psychometric properties is warranted. Fourth, recall errors and biases of social desirability can influence our results as patients may positively report their health behaviors^49^ and perceptions. Lastly, our study recruited patients from multiple disease sites, treatment intents, and types of therapies received which may lead to possible confounding by these factors. For example, patients with tumors from different sites may have received different levels of counselling on smoking, PA, or alcohol consumption. However, this heterogeneity has its own advantage given the strength of the associations identified in this population. We plan to validate our findings in future studies focused on specific disease site populations.

In summary, we have identified that immigrant cancer survivors were less likely to be aware of the harms of continued smoking and benefits of PA. Non‐Western survivors were less likely to smoke at baseline, and both immigrants and non‐Western survivors were less likely to meet PA guidelines at baseline or consume alcohol at diagnosis. Immigrants may face numerous unique barriers and require consideration for culturally tailored health behavior modification strategies. Future studies should focus on strategies to implement survivorship programs to help improve the health behaviors of immigrant cancer survivors.

## CONFLICT OF INTEREST

The authors have declared no conflicts of interest.
